# There Is No Carbon Transfer Between Scots Pine and Pine Mistletoe but the Assimilation Capacity of the Hemiparasite Is Constrained by Host Water Use Under Dry Conditions

**DOI:** 10.3389/fpls.2022.902705

**Published:** 2022-05-26

**Authors:** Ao Wang, Marco M. Lehmann, Andreas Rigling, Arthur Gessler, Matthias Saurer, Zhong Du, Mai-He Li

**Affiliations:** ^1^Forest Dynamics, Swiss Federal Institute for Forest, Snow and Landscape Research WSL, Birmensdorf, Switzerland; ^2^Institute of Terrestrial Ecosystems, ETH Zürich, Zurich, Switzerland; ^3^School of Geographical Sciences, China West Normal University, Nanchong, China

**Keywords:** *Viscum album* ssp. *austriacum*, *Pinus sylvestris*, ^13^C assimilates, non-structural carbohydrate (NSC), host water, carbon relationship

## Abstract

Pine mistletoe is a hemiparasitic shrub that can produce its own photosynthates. There is a lack of knowledge about the interaction of mistletoe and host under varying environmental condition that might influence carbon gain and allocation. In a ^13^C-pulse labeling experiment with mature *Pinus sylvestris* (pine) infected by mistletoes grown in naturally dry or irrigated conditions, (1) mistletoe clusters were shielded from ^13^CO_2_ added, and (2) mistletoes or host needles were removed to manipulate the local assimilate and water availability. No ^13^C signal was found in shielded mistletoes, indicating no carbon transfer from the host to the mistletoe. When the pine needles were removed from girdled branches, no ^13^C signal was found in the host tissues, implying no carbon transfer from mistletoe to the host. However, mistletoes on needle-removed pine trees accumulated more labeled assimilates and had higher non-structural carbohydrate (NSC) concentrations only under naturally dry conditions but not in irrigated plots. Our results suggest that mistletoes show full carbon autonomy, as they neither receive carbon from nor provide carbon resource to the host trees. Moreover, the high assimilation capacity of mistletoes seems to be constrained by the host water use under dry conditions, suggesting that drought stress is not only negatively impacting trees but also mistletoes. Therefore, we conclude that the hemiparasites live on their own in terms of carbon gain which, however, depends on the water provided by the host tree.

## Introduction

The relationship between a parasite and its host is important ecologically and widely discussed in animal and plant pathology and physiology. Most research on parasite–host relationships in plants has concentrated on host responses to infections by parasites (Streicker et al., 2013; Solomon et al., 2015). In contrast, the interactions between plant hosts and plant parasites, especially the effects of hosts on parasites in different habitats and varying site conditions, have rarely been studied. However, such parasite–host relationships, including a possible feedback system between the host and parasite, are of central interest because they can strongly affect the growth and survival of the higher plants serve as hosts.

Mistletoes are well-known hemiparasitic plants that maintain their own carbon assimilation by photosynthesis and can infect many tree species in various ecosystem types worldwide, making them an important and relevant species in parasitism research (Zuber, 2004; Glatzel and Geils, 2009). *Viscum album* ssp. *austriacum* (Santalaceae), pine mistletoe, is the most widely distributed species across the European continent (Zuber, 2004; Dobbertin and Rigling, 2006). Pine mistletoe survival and development in forest ecosystems mainly rely on water and mineral resources obtained from the xylem sap of the host tree (Dobbertin and Rigling, 2006; Rigling et al., 2010). If water availability is high and nutrients are not limited, pine mistletoes and their hosts co-exist for years without major restrictions for the host tree (Zuber, 2004; Solomon et al., 2015). However, if water is limited during dry periods, the high-water consumption and low water-use efficiency of pine mistletoes may exacerbate drought stress in the host tree, with negative consequences on the host’s physiology and growth performance (Dobbertin and Rigling, 2006; Rigling et al., 2010; Zweifel et al., 2012). As a consequence, pine mistletoe infection leads to a reduction of branching and of branch and needle growth (Rigling et al., 2010), resulting in an increased risk of mortality for the host tree (Dobbertin and Rigling, 2006). This contribution of pine mistletoe to drought-induced forest decline processes has been demonstrated in several xeric forest ecosystems in Spain (Galiano et al., 2011; Sangüesa-Barreda et al., 2012, 2013; Scalon et al., 2013) and inner-Alpine regions in Switzerland and Italy (Dobbertin and Rigling, 2006; Rigling et al., 2010; Vacchiano et al., 2012).

Along with the negative effects of mistletoes on the host water balance, pine mistletoes have also been found to affect the carbon balance of the host in a variety of ways (Glatzel and Geils, 2009; Scalon and Wright, 2015; Le et al., 2016b). High water use of mistletoes and thus considerable water loss from the whole host–parasite system may induce closure of the stomata in host trees to save water (Rigling et al., 2010; Zweifel et al., 2012), resulting in lower photosynthesis rates of the trees (Dobbertin and Rigling, 2006; Yan et al., 2016). Therefore, pine mistletoe can indirectly reduce the host’s ability to acquire carbon resources, especially under drought-stress conditions (Sangüesa-Barreda et al., 2013; Yan et al., 2016).

Mistletoes perform photosynthesis at a rate similar to that of the host (Lüttge et al., 1998; Scalon and Wright, 2017). In some studies, however, it has been reported that mistletoes are able to additionally acquire organic carbon from the host in the form of xylem-mobile organic acids and amino acids (Escher et al., 2004b; Těšitel et al., 2010). Richter et al. (1995) estimated that mistletoe leaves take up over 50% of its required heterotrophic carbon from its host. Nevertheless, according to Smith and Gledhill (1983), the haustorium of *V. album* grows only within the host’s xylem and does not connect to the host’s phloem. This means that there should be only acropetal carbon transport from the host xylem to the mistletoe via the transpiration stream, with no basipetal carbon flow from the mistletoe to the host, even under strong carbon limitation of the host (Glatzel and Geils, 2009; Scalon and Wright, 2015). Hence, it remains unclear whether mistletoes can directly absorb carbon resources from host tissues in considerable amounts, in addition to their own photosynthetic activities. By utilizing the stable ^13^C isotope tracer technique, it is possible to determine the direction and quantity of carbon assimilate flow between mistletoe and host, and also to assess how this process depends on carbon and water availability.

Most studies on mistletoe–host relationships have been conducted by comparing trees infected by mistletoes with non-infected trees growing under the same conditions (Dobbertin and Rigling, 2006; Dobbertin et al., 2010; Rigling et al., 2010; Yan et al., 2016). Whether the host’s carbon resource availability, which is strongly associated with its growth conditions (e.g., soil water moisture), affects the mistletoe–host relationship has only been investigated in a few studies, and these studies were only focused on the response of hosts to mistletoe infection (Zweifel et al., 2012; Sangüesa-Barreda et al., 2013; Le et al., 2016a). It is still unclear if the carbon dynamics in the mistletoe and in its host, as well as the potential exchange of assimilates between the two, changes in response to the local water availability of both the host tree and the mistletoe.

To address these unresolved questions, we conducted two separate experiments under the umbrella of a whole-tree ^13^C-pulse labeling experiment with mature Scots pine (*Pinus sylvestris*) trees infected by pine mistletoe (*V. album*). Host trees whose crowns were exposed to ^13^CO_2_ were growing either in naturally dry conditions (∼600 mm precipitation per year) or in irrigated areas (+ 700 mm per year, applied during the growing season) for 15 years in the Swiss Pfynwald forest ecosystem experimental platform (Schaub et al., 2016; Joseph et al., 2020).

In a wrapping experiment (Exp. 1), we shielded mistletoe clusters with gas-tight plastic foil and darkened them with aluminum foil before the whole-tree labeling to prevent ^13^C assimilation by these clusters. We investigated the ^13^C values in both wrapped and non-wrapped mistletoes, as well as in their host twigs, to test the hypothesis (**H1**) that *V. album* takes up carbon resources from its host via the haustorium. Any signal in the wrapped mistletoes (shielding from ^13^CO_2_ and light exclusion) would originate from the host and we assumed the contribution of the host (if any) to be higher in irrigated vs. drought-stressed trees due to increased assimilation rates in irrigated trees (Schonbeck et al., 2021).

To change source–sink carbon and water relationships, we performed a tissue removal experiment (Exp. 2). We girdled pine branches infected with mistletoes of drought-stressed and irrigated host trees to restrict the phloem carbon translocation between the remaining tree and the girdled branch (Andersen et al., 2005; De Schepper and Steppe, 2013), while keeping a constant water and nutrient flow. Beyond the girdling point, we then removed all pine needles or all mistletoe tissues (including stem and leaves) from the girdled pine branches to manipulate source-sink relationships and water relations locally on the branch level. Through Exp. 2, we aimed to test the hypothesis (**H2a**) that local changes in source–sink relationships by reducing assimilate ability (i.e., host needle removal), would decrease the mistletoes’ carbon level due to lower amounts of carbon obtained from the host (conditional H1 is supported). An alternative hypothesis (**H2b**) is that needle removal increases the mistletoes’ carbon level due to increased carbon assimilation by the hemiparasite itself as a result of decreased competition for water with the host. This effect would be more pronounced under the dry control conditions. Finally, we hypothesize (**H3**) that mistletoes do not provide any carbon to the host, even when the host is carbon limited due to needle removal.

## Materials and Methods

### Study Site

Our experiment was conducted in a naturally regenerated, mature Scots pine (*Pinus sylvestris*) forest in Pfynwald, Valais, Switzerland (46°19′27″N, 7°34′40″E, 610 m a.s.l.). The forest site is located in a dry, inner-Alpine region of Switzerland with repeated occurrence of Scots pine mortality events within the last decades (Dobbertin et al., 2005; Rigling et al., 2013). The mean annual precipitation is around 657 mm and the mean annual temperature is 9.7°C (Dobbertin et al., 2010). A long-term irrigation experiment was started in 2003, where four plots of 1,000 m^2^ are irrigated with water from a nearby channel during the growing season (+ 700 mm year^–1^, resulting in 1,300–1,400 mm total precipitation per year = irrigated). Four additional plots of the same size are used as a naturally dry control (= non-irrigated). The dominant species in this forest (> 10 km^2^) is *P. sylvestris*, with *Quercus pubescens* occurring occasionally. The pine trees are over 100 years old, with a mean height of ∼11 m and a diameter at breast height (DBH) of ∼12 cm (Schaub et al., 2016). The soil type is a Rendzic Leptosol derived from limestone (Brunner et al., 2009). Many of the Scots pine trees are severely infected by pine mistletoe (*V. album*), with variations in the density and age of the mistletoes (mostly more than 10 years old) (Dobbertin et al., 2010). Since the irrigation experiment started, the environmental conditions (i.e., air temperature, air humidity, precipitation, soil temperature, and soil water potential) have been continuously monitored.

### ^13^C Labeling at the Whole-Tree Level

**FIGURE 1:**
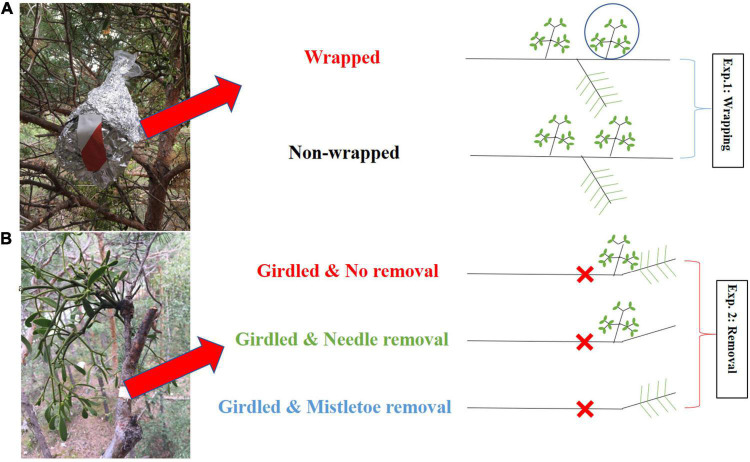
Scheme of the wrapping experiment [**(A)**: Exp. 1] and the girdling and removal experiment [**(B)**: Exp. 2], with drawings showing the treatments applied to mistletoes and pine twigs on the right-hand site. In Exp. 1, mistletoe clusters were covered with gas-tight plastic sheets and aluminum foil to prevent direct ^13^C label uptake during the whole-tree ^13^CO_2_ labeling experiment; non-wrapped controls were allowed to take up ^13^CO_2_. In Exp. 2, the bark including phloem was girdled for three branches per tree before the whole-tree labeling to create an isolated environment without top-down carbon transportation via the host’s phloem, and each of these three branches was randomly assigned to mistletoe removal, pine needle removal, or intact control.

We conducted whole-tree-crown ^13^C labeling experiments in summer 2017, i.e., in the 15th year of irrigation treatment (Joseph et al., 2020). Six mature pine trees that were severely infected (over) by mistletoes (three control and three irrigated) were selected from the labeling experiment for the present study. For each selected tree, the whole tree crown was enclosed within a large temperature controlled transparent chamber, and approx. 10 g of CO_2_ with > 99 atom% ^13^C (Cambridge Isotopes, Tewksbury, MA, United States) was released into the chamber over a period of 3.5 h. Pulse labeling of the six trees was applied from 29 to 31 August 2017 (one pair of trees (control/irrigated) per day) (Supplementary Figure 1). Within this whole-tree labeling experiment, we conducted the following two experiments for the present study (Figure 1).

#### Wrapping Experiment (Exp. 1)

We selected six to eight mistletoe clusters from each of the six ^13^C-labeled trees. Half of them (three to four) were randomly selected and wrapped with gas-tight plastic sheets to avoid direct uptake and assimilation of ^13^CO_2_, and additionally darkened with aluminum foil to avoid exposure to sunlight before the chamber was closed (Figure 1). The other three to four clusters selected per tree remained unwrapped and were allowed to take up ^13^CO_2_ during the 3.5 h of labeling. We did not cover pine needles with gas-tight plastic sheets and aluminum foil because we expected that *V. album* takes up carbon resources with increased ^13^C signal labeled from its host via the haustorium, but not vice versa (see our hypothesis H1). Tissues from the host (needle, twig xylem and phloem) and mistletoe clusters (leaf and shoot) were sampled at –1 day (the day before labeling) and at 4, 8, 24 h, 3, 7, 14, 30, 60, and 180 days after the start of labeling for analysis of the ^13^C abundance.

#### Removal Experiment (Exp. 2)

In each of the six ^13^C labeled trees considered in this study, three well-foliaged branches (around 1 m in length) that were infected by mistletoes were selected for a girdling treatment (Figure 1). A bark strip (including the phloem) of 2 cm width was removed over the entire circumference of the branch, basipetal to the mistletoe, to stop the basipetal transport of photo assimilates but to keep the xylem intact for the upward water and nutrient transport (Figure 1), 1 day before the whole-tree labeling started. In one of the three girdled branches per tree, all mistletoe tissues (leaf and shoot) were completely removed from the host branch at the point of injection with scissors before the ^13^CO_2_-labeling (mistletoe removal), while in a second girdled branch, all host needles were easily removed from the host branch by bare hand (needle removal). The third one was kept intact and was used as a control (no removal). Plant material from the host (needle, twig xylem and phloem) and mistletoe (leaf and shoot) were sampled at –1 day (the day before labeling) and at 4, 8, 24 h, 3, 8, and 15 days after the start of labeling for ^13^C abundance measurements. Measurement of non-structural carbohydrate (NSC) concentrations was conducted for the sampling at –1 day, label 1, 3, 8, and 15 days.

### Analysis of Morphological Traits in Pine Needles and Mistletoe Leaves

One day before the labeling, 10 mistletoe leaves and 20 pine needles of each selected tree were harvested separately for leaf morphological measurements. Leaf area was measured using a scanner and image analysis software (PIXSTAT v1.3, WSL, Birmensdorf, Switzerland). Fresh weight of all leaves was firstly measured, and dry weight was measured after oven-drying the samples at 65°C for 5 days, and leaf water content on a fresh weight basis and leaf dry mass per unit leaf area (LMA) were then calculated.

### Analysis of Non-structural Carbohydrate Concentrations

All tissues harvested for NSC and isotope analyses were dried in an oven at 65°C for 5 days. After drying, each sample was ground with a Retsch MM 300 ball mill (Retsch, Germany) until finely and homogeneously ground. NSCs are defined here as low-molecular-weight sugars and starch, and analysis followed the protocol by Schönbeck et al. (2018). About 10 mg of the sample powder was first vortexed with 2 ml of deionized water and then boiled in the steam for 30 min. For free-sugar analysis, a 200 μl aliquot of the extract was treated with invertase and isomerase (in 0.4 M Na-acetate buffer; Sigma-Aldrich, St. Louis, MO, United States) to break down sucrose to fructose and glucose. For the total NSC_*T*_ (NSC_*T*_ = soluble sugars + starch) analysis, a 500 μl aliquot of the extract (sugars and starch) was incubated with a fungal amyloglucosidase from *Aspergillus niger* (Sigma-Aldrich, St Louis, MO, United States) for 15 h at 49°C to digest starch into glucose. Both soluble sugars and NSC_*T*_ concentrations were determined at 340 nm in a 96-well microplate photometer (Multiskan GO, Thermo Fisher Scientific, Waltham, MA, United States) after enzymatic conversion of glucose molecules derived from sugars and starch to gluconate-6-phosphate (via isomerase, hexokinase, and glucose-6-P dehydrogenase; all supplied by Sigma-Aldrich). NSC concentrations are expressed as a percentage of dry matter, and the concentration of starch was calculated as NSC_*T*_ minus free sugars.

### Analysis of ^13^C Abundance

Around 1 mg of ground tissue material (same as used for the NSC analysis) was weighed into tin capsules. Organic carbon was converted to CO_2_ in an elemental analyzer Euro EA3000 (Hekatech GmbH, Wegberg, Germany) connected to an isotope ratio mass spectrometer (IRMS; Delta V Advantage, Thermo Fisher Scientific, Bremen, Germany) to determine the total carbon and carbon isotopic composition. Laboratory standards with known ð^13^C values were measured with a precision of 0.1‰. The isotopic ratios in all samples were expressed in δ notation (‰) relative to the international standard Vienna Pee Dee Belemnite (VPDB). The carbon isotope ratio was corrected to account for pre-labeling isotope ratios of bulk material to indicate the extent of ^13^C-label incorporation in different tissues.


(1) ΔδC13=δC13-LδC13AN

where δ^13^C_*L*_ is the isotope ratio after the start of the labeling and δ^13^C_*NA*_ is the natural (pre-labeling) isotope abundance.

### Data Analysis

All data (i.e., δ^13^C, NSC_*T*_ and its components) were first tested for normality with Kolmogorov–Smirnov tests to assess the within- and between-subject effects in different tissues of pine (i.e., needle, xylem, phloem) and mistletoe (i.e., leaf, shoot).

For the wrapping experiment (Exp. 1), a linear mixed model (tree replicates as random effect) was used for testing the effects of time (sampling time), irrigation treatment (i.e., non-irrigated vs. irrigated), wrapping treatment (i.e., wrapping vs. non-wrapped), and their interactions on the carbon isotopic composition in different mistletoe tissues (i.e., leaf, shoot). The assessment of residuals normality and homoscedasticity were tested before analysis.

For the removal experiment (Exp. 2) (above the girdled branches), the assessment of residuals normality and homoscedasticity were tested before analysis. A linear mixed model (tree replicates as random effect) was used for testing the effects of time (sampling time), irrigation treatment (i.e., non-irrigated vs. irrigated), removal treatments (i.e., needle removal for phloem, xylem and mistletoe tissues; mistletoe removal for pine tissues; and no removal for both pine and mistletoe tissues), and their interactions on δ^13^C and NSC_*T*_ concentrations. For each sampling time point, one-way ANOVA and a Tukey-HSD *post hoc* test were used to investigate the difference in δ^13^C and NSC_*T*_ concentrations in different pine and mistletoe tissues under different irrigation and removal treatments. R version 4.1.0 was used for all statistical analyses (R Core Team, 2021).

## Results

### Irrigation Effects on the Morphology of Pine Needles and Mistletoe Leaves

**FIGURE 2:**
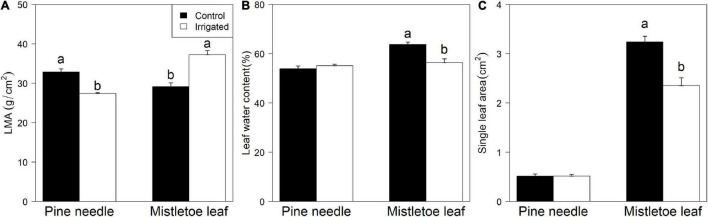
Comparison of the leaf traits between *Pinus sylvestris* and *Viscum album* ssp. *austriacum* in the control and irrigation treatments (*n* = 3 trees per treatment). **(A)** Leaf mass per unit leaf area (LMA) of mistletoe leaves and pine needles, **(B)** leaf water content of mistletoe leaves and pine needles, **(C)** single leaf area of mistletoe leaves and pine needles. Different letters indicate significant differences (*P* < 0.05) for each tissue between dry controls and irrigated trees.

Long-term irrigation significantly influenced the morphological traits of both pine needles and mistletoe leaves. Host leaf mass per unit leaf area (LMA) was significantly higher in control trees than in irrigated ones, but mistletoe leaves showed the opposite pattern (Figure 2A). There was no detectable difference in leaf water content between irrigated and control pine needles (Figure 2B). In contrast, leaf water content was significantly higher in control compared with irrigated mistletoe leaves (Figure 2B). The area of single pine needles was not affected by the irrigation treatment, but mistletoe had significantly larger leaves under control dry conditions than when irrigated (Figure 2C).

### Wrapping Effects on ^13^C Assimilates in Mistletoe Tissues (Exp. 1: Wrapping)

**FIGURE 3:**
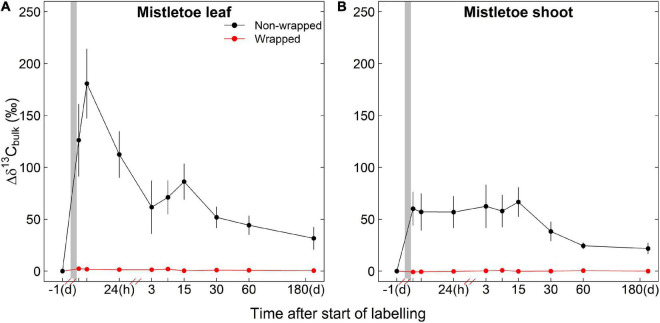
Incorporation of the ^13^C label into the bulk organic matter (Δδ^13^C_*bulk*_) of mistletoe leaves **(A)** and shoots **(B)** in the wrapping experiment (i.e., wrapped vs. non-wrapped; *n* = 3) after a 3.5 h of exposure to ^13^C-enriched CO_2_ (gray shaded area). Please note that the initial point is from 1 day before the labeling and that the scaling of the *x*-axis changes after each break.

Irrigation had no significant effects on the ^13^C assimilates of mistletoe tissues in the wrapping experiment, whereas the wrapping treatment had a strong effect on the ^13^C accumulation in mistletoe tissues (Table 1). Strong labeling signals were found in the non-wrapped mistletoe leaves and shoots, while in the wrapped mistletoe clusters no ^13^C signal was found in leaves or shoots (Figure 3). The peak value of Δδ^13^C in non-wrapped leaves occurred approximately 8 h after the labeling started, after which point Δδ^13^C values decreased gradually (Figure 3A). Δδ^13^C values reached a peak in wrapped mistletoe shoots at the first sampling time after labeling, remained at a relatively stable high level until 15 days after labeling, and decreased slowly thereafter (Figure 3B).

**TABLE 1:** Results of linear mixed models for Δδ^13^C values (uptake and incorporation of ^13^C) of bulk material in different tissues of *Viscum album* ssp. *austriacum* in the wrapping experiment (Exp. 1).

Factors	df	Mistletoe leaf Δδ^13^C	Mistletoe shoot Δδ^13^C
Time (T)	9	9.08***	3.43**
Irrigation (I)	1	1.11	0.47
Wrapping (W)	1	185.99***	137.74***
T × I	9	1.99	1.03
T × W	9	8.70***	3.49**
I × W	1	2.72	3.41
T × I × W	9	1.80	0.47

**P < 0.01, ***P < 0.001.

*Degrees of freedom (df) and F-values are given for time, irrigation treatment and wrapping treatment (i.e., wrapped vs. non-wrapped; n = 3).*

### Removal Effects on Carbon Assimilates in Pine and Mistletoe Tissues (Exp. 2: Girdling and Removal)

**FIGURE 4:**
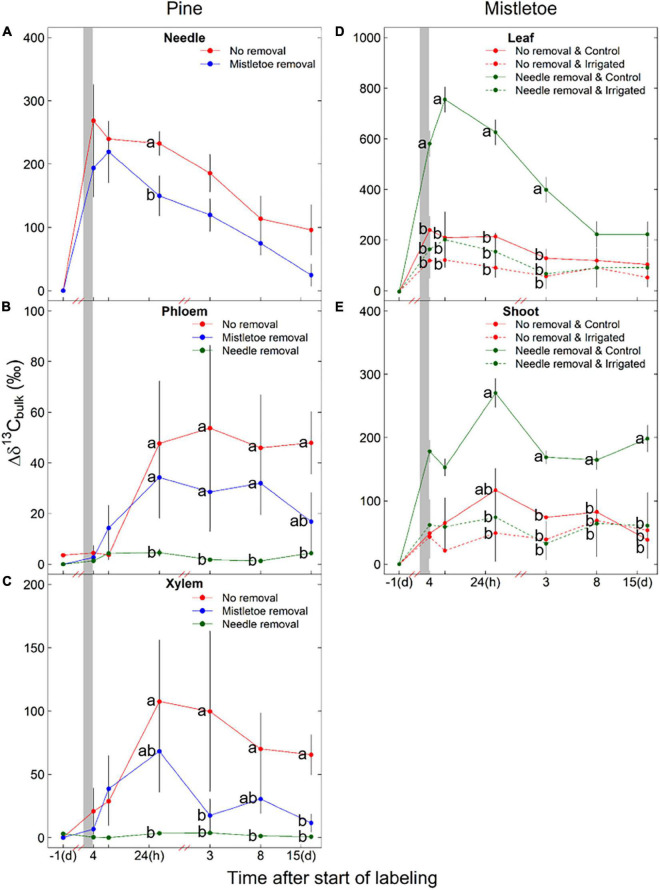
Initial (1 day before the labeling) and incorporation of the ^13^C label into the bulk organic matter (Δδ^13^C_*bulk*_) of different pine (*Pinus sylvestris*) **(A–C)** and mistletoe (*Viscum album* ssp. *austriacum*) **(D,E)** tissues under different removal treatments (i.e., pine needle removal for pine phloem and xylem, mistletoe leaf and shoot; mistletoe removal for pine needle, phloem and xylem). Girdled branches were exposed to ^13^C-enriched CO_2_ (shaded area) for 3.5 h. Dashed lines **(D,E)** are used to indicate where the Δδ^13^C values of mistletoe leaves and shoots showed a significant difference (*P* < 0.05) between irrigated and non-irrigated trees (see Table 2). Different letters indicate significant differences among treatments at the same sampling time (*n* = 3). Please note the difference in scale of the *x*-axis before and after the red break points and the difference in *y*-axis scale among panels.

**FIGURE 5:**
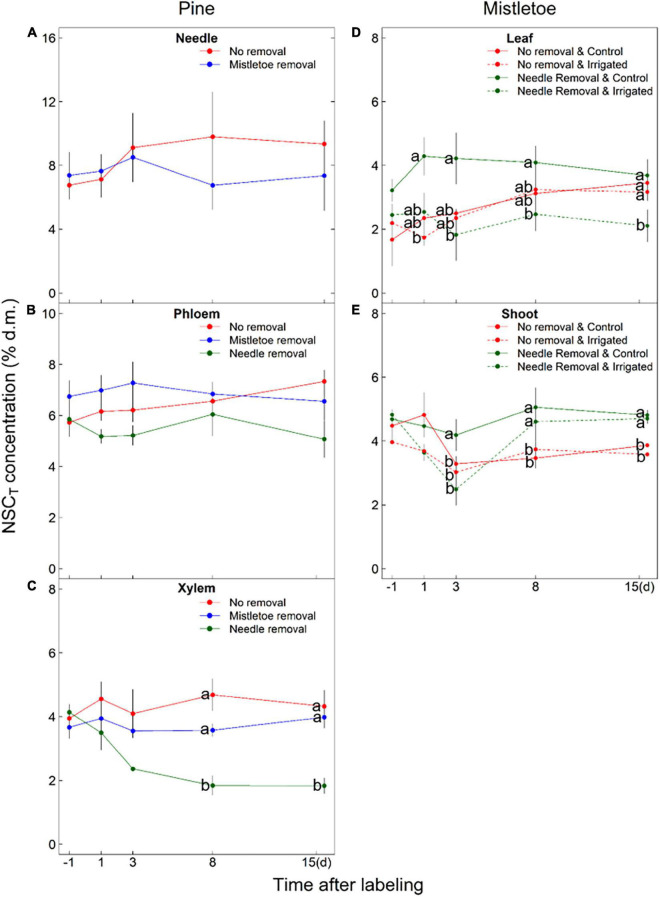
NSC_*T*_ concentration [% of dry matter (d.m.)] in different pine (*Pinus sylvestris*) **(A–C)** and mistletoe (*Viscum album* ssp. *austriacum*) **(D,E)** tissues under different removal treatments (i.e., pine needle removal for pine phloem and xylem, mistletoe leaf and shoot; mistletoe removal for pine needle, phloem and xylem) applied to the girdled branches. The initial samples were collected 1 day before the labeling. Dashed lines **(D,E)** are used to indicate where the irrigation treatment had significant effect (*P* < 0.05) on NSC_*T*_ concentration (see Table 2). Different letters indicate significant differences among treatments at the same sampling time (*n* = 3). Please note the difference in *y-*axis scale among panels.

The irrigation treatment and its interaction with other factors (time or removal treatment) did not affect Δδ^13^C or NSC concentration (NSC_*T*_, sugars, starch) in the pine tissues (Table 2). However, there was a direct effect of the removal treatments on the carbon assimilates in host tissues (Table 2). Mistletoe removal resulted in significantly lower needle Δδ^13^C at 24 h after labeling (Figure 4A), but did not affect needle NSC concentrations (Table 2 and Figure 5A). Mistletoe removal did not affect new carbon assimilates in the host phloem (Figures 4B, 5B) but led to significantly lower Δδ^13^C in the pine xylem tissue at 3 and 15 days after labeling (Figures 4C, 5C). When the needles were removed, no significant ^13^C signals were found anymore in host phloem or xylem (Figures 4B,C). Needle and mistletoe removal did not affect the NSC concentrations in pine phloem (Figure 5B). Needle removal decreased the host xylem NSC concentrations, while such effects were not observed in the mistletoe removal treatment (Figure 5C).

**TABLE 2:** Results of linear mixed models for Δδ^13^C values (uptake and incorporation of ^13^C) of bulk material, as well as the concentration of NSC_*T*_ and its compounds (i.e., soluble sugars and starch) in different tissues of *Pinus sylvestris* and *Viscum album* ssp. *austriacum* in the removal experiment (Exp. 2).

	df	Δδ^13^C	df	NSC_*T*_	Sugar	Starch
**Pine needle**						
Time (T)	6	18.25***	4	0.89	0.14	0.87
Irrigation (I)	1	0.11	1	0.06	1.01	0.01
Mistletoe removal (MR)	1	9.94***m	1	2.11	2.64	0.38
T × I	6	0.58	4	0.54	0.72	0.89
T × R	6	1.13	4	1.31	1.54	0.96
I × MR	1	0.43	1	0.11	0.36	1.02
**Pine phloem**						
Time (T)	6	3.26*	4	0.33	1.99	1.93
Irrigation (I)	1	0.17	1	1.40	0.01	2.61
Removal (R)	2	10.23***	2	11.35***	6.49*	5.39*
T × I	6	0.14	4	1.59	1.22	1.08
T × R	12	1.15	8	1.23	1.66	1.74
I × R	2	0.48	2	0.31	1.73	1.38
**Pine xylem**						
Time (T)	6	2.29*	4	2.36	2.06	1.71
Irrigation (I)	1	0.08	1	0.63	1.21	2.21
Removal (R)	2	11.89***	2	23.58***	21.93***	9.87**
T × I	6	0.14	4	1.20	1.51	0.89
T × R	12	1.13	8	3.25**	2.63*	2.95*
I × R	2	0.06	2	2.04	1.11	0.84
**Mistletoe leaf**						
Time (T)	6	9.24***	4	2.71	0.44	2.28
Irrigation (I)	1	11.89***	1	5.14^†^	0.59	3.04^†^
Needle removal (NR)	1	25.93***	1	7.69*	5.23*	6.01*
T × I	6	10.35***	4	1.22	1.02	0.87
T × NR	6	8.21***	4	2.83^†^	2.54^†^	2.77^†^
I * NR	1	15.47***	1	17.62***	14.54***	11.25***
**Mistletoe shoot**						
Time (T)	6	16.03***	4	2.41	0.44	1.88
Irrigation (I)	1	4.45^†^	1	3.74^†^	2.05	3.03^†^
Needle removal (NR)	1	25.11***	1	9.08*	4.72*	4.09*
T × I	6	4.11**	4	1.13	1.17	1.24
T × NR	6	2.62*	4	0.93	0.87	0.74
I * NR	1	35.15***	1	0.12	0.05	2.64

*P < 0.05, **P < 0.01, ***P < 0.001, ^†^P < 0.1. The degrees of freedom (df) and F-values are given (P-values are given with the significance level indicated, with values corresponding to P < 0.05 given in red) for time, irrigation treatment and different removal treatments (i.e., mistletoe removal for pine needle, needle removal and mistletoe removal for pine phloem, and xylem, needle removal for mistletoe leaf and shoot; n = 3).

Irrigation significantly (*P* < 0.05) or marginally significantly (*P* < 0.10) influenced the Δδ^13^C and NSC concentrations (except sugars) in both mistletoe leaves and shoots (Table 2), and host needle removal also significantly affected the carbon assimilates and NSC concentrations in both mistletoe leaves and shoots (Table 2). In addition, host needle removal interacted with irrigation to significantly affect both Δδ^13^C and NSC concentrations in mistletoe leaves and only Δδ^13^C in mistletoe shoots (Table 2). Mistletoe leaves and shoots on trees grown under dry control conditions tended to have higher Δδ^13^C levels than those on irrigated trees, especially when the host needles were removed (Figures 4D,E). Similarly, NSC concentrations in mistletoe leaves were higher in the control compared with the irrigated trees, but mainly when the pine needles were removed (Figures 5D,E).

## Discussion

### No Transport of Newly Assimilated Carbon From Host to Mistletoes

The wrapping experiment showed that non-wrapped mistletoes efficiently assimilate carbon (Figure 3), although the incorporation of ^13^C originating from the labeled CO_2_ in mistletoe leaves was only half of that in pine needles (Figures 3, 4). We did not find a strong effect of irrigation on the ^13^C incorporation in mistletoe leaves or shoots (Table 1), indicating that the carbon assimilation capacity of mistletoes was not affected by restricted soil water availability, although mistletoes are known to rely on acquiring water resources via the xylem of the host tree (Glatzel and Geils, 2009; Zweifel et al., 2012). Scalon and Wright (2017) investigated 42 mistletoe–host species pairs sampled from 5 sites in Australia and Brazil under different soil water availability and found that the photosynthetic capacity of mistletoes and their hosts were on a similar level, but that mistletoes had leaf dark respiration rates that were twice that of the hosts at a given photosynthetic capacity, resulting in higher leaf maintenance costs for these hemiparasitic plants. In our study, it is possible that higher respiration rates, and thus loss of previously fixed ^13^C, contributed to the lower overall incorporation of ^13^C in bulk organic matter of mistletoe leaves compared with pine needles.

In contrast, wrapped mistletoes were not able to assimilate new carbon assimilates after the labeling event (Figure 3). This clearly shows that new carbon assimilates are not transported from the host to any mistletoe tissue in significant amounts, which is consistent with the concept suggested in previous studies assuming that no phloem connection is established between hemiparasite and host (Glatzel and Geils, 2009; Těšitel et al., 2010; Scalon and Wright, 2015). In contrast, Marshall and Ehleringer (1990) compared the nature abundance of ^13^C values measured with gas exchange measurement-based theoretical ^13^C values in mistletoe leaves, and found that only part of the carbon in the biomass of *V. album* originated from its own photosynthesis activities. Escher et al. (2004a) speculated that pine mistletoes gained heterotrophic carbon from the host via the xylem sap, based on significantly positive correlations of soluble carbohydrate between mistletoe and host. However, such “extra” carbon gain of mistletoe derived from xylem (i.e., from needles, downwards to roots, and then upwards to xylem) was not evidenced by our labeling experiment. Even after 180 days, we did not find any ^13^C signal in the wrapped mistletoe clusters, indicating that, in the longer term, no labeled carbon was obtained from the host. Within the same whole-tree labeling experiment, Gao et al. (2021) showed that even 10 months after labeling, tree’s respired CO_2_ still had δ^13^C values of up to 25‰. This indicates that significant amounts of label were still present in the host’s tissues and transport systems on our final sampling date, yet not transferred to the mistletoes. We conclude that mistletoes are complete carbon autotrophs and do not receive significant amounts of carbon directly from the host; thus, we can reject **H1**.

### The Photosynthetic Capacity of Pine Mistletoes Is Suppressed by the Host Trees Under Drier Conditions

Although we did not find a significant effect of the irrigation treatment on mistletoe ^13^C uptake and incorporation in the wrapping experiment (Figure 3), mistletoe leaves and shoots accumulated more ^13^C-labeled assimilates in trees grown in the dry control conditions than in the irrigated conditions, when the needles of trees in both conditions were removed from the girdled branches (Table 2 and Figure 4). As photosynthetic carbon acquisition is normally greater in an environment with higher soil moisture (Wang et al., 1998; Reich et al., 2018; Joseph et al., 2020), our results may be attributed to an increased water supply to mistletoes due to reduced water use by needle removal in the dry conditions which had significantly lower soil moisture than the irrigated plots (Supplementary Figure 2). Such an abrupt water release may result in a short-term pulse effect (<3 days; Figure 4D) of water on the photosynthetic capacity and thus ^13^C levels in mistletoe leaves (Figure 4D). This may imply that mistletoes compete for water with the host in dry but not in wetter conditions, as the former had significantly lower soil water moisture showing possible water limitation while the latter had higher soil moisture remaining near optimal water conditions (Supplementary Figure 2). Similarly, Zweifel et al. (2012) found that the stomatal conductance of pine needles was significantly negatively correlated with the levels of mistletoe infection in *P. sylvestris* trees grown in conditions with ∼600 mm precipitation, suggesting water competition between mistletoe and its host in dry environment. Moreover, mistletoe leaves were larger and had a higher water content under the dry control conditions (Figure 2), and thus may have a higher photosynthetic potential, leading to increased ^13^C signal (Figures 4D,E). Similarly, previous studies suggested that changes in host water and nutrient condition can regulate the water and carbon uptake efficiency of a hemiparasitic mistletoe (*Phoradendron juniperinum*) (Marshall et al., 1994), and a holoparasitic mistletoe (*Arceuthobium vaginatum* subsp. *Cryptopodum*) (Bickford et al., 2005). These results further signify that host needle removal not only affected source–sink carbon relationships in the girdled branch but might also result in more available water for the mistletoe due to discontinued host transpiration. The reduced competition for water may allow the mistletoes to keep stomates more open, thus allowing for higher photosynthesis rates.

We also found that mistletoe leaf NSC_*T*_ concentrations were significantly higher in the control trees compared with the irrigated trees when pine needles were not present anymore (Table 2 and Figure 5), which corroborates our assumption of higher assimilation by mistletoes when there were no needles to demand water under dry conditions. The high carbon accumulation capacity of mistletoes in a stressful environment also demonstrates the competitive ability of the hemiparasite. However, it seemed that mistletoes with bigger leaf size and higher assimilation potential benefit more from pine needle removal than the pine needles benefit from mistletoe removal (see discussion below). Still, mistletoes are known to exacerbate tree mortality in drought-exposed regions (Dobbertin and Rigling, 2006; Rigling et al., 2010; Zweifel et al., 2012; Sangüesa-Barreda et al., 2013; Durand-Gillmann et al., 2014). Our findings support our hypothesis **H2b** that expected an increased carbon level in mistletoes due to increased carbon assimilation by the hemiparasite itself as a result of decreased competition for water with the host after host needle removal. In contrast to our hypothesis **H2a**, an increase in carbon level and carbon assimilation of mistletoes after host needle removal was found, which is not related to a change in source activity of the host but is rather due to the released water restrictions of the mistletoe by removing the transpiring host needles under dry conditions.

### Mistletoes Cannot Act as a Carbon Provider, Even When the Host Is Carbon Limited

In the needle removal treatment, no strong ^13^C signal was found upon ^13^C labeling in the pine sink tissues (i.e., phloem and xylem). The minimal ^13^C traces detected (Figures 4B,C) might be due to bark photosynthesis (Aschan and Pfanz, 2003; Simkin et al., 2020). This finding suggests that mistletoe assimilates do not act as a significant carbon source for the host sink tissues. We thus conclude that there is no exchange of carbon assimilates between mistletoe and host. Neither do mistletoes use host carbon resources nor do they provide any carbon to the host, even when the host is carbon limited in special situations (needle removal treatment, drought). These results support **H3** and prove that mistletoes and hosts are carbon-independent and that only water and nutrients are transported from the host to the mistletoes.

Meanwhile, needle removal also resulted in a decrease in NSC_*T*_ concentrations in pine xylem tissue after 1 week, which can be explained by the lack of delivery of new assimilates to the sink tissues. Pine xylem, as well as needles and phloem, however, also tended to accumulate less ^13^C-labeled carbon assimilates when mistletoes were removed from the branches (Figures 4A–C). Since there is no transport of assimilates from the mistletoe to the host tissues, we propose the following explanation: mistletoe tissues have lower water potentials compared with host tissues, which ensures continuous water uptake from the host xylem (Schulze et al., 1984; Zweifel et al., 2012; Scalon and Wright, 2015), and continuously compete for water with pine tissues. Removing the mistletoe might reduce the need to incorporate large amounts of osmotically active compounds, and thus decrease the transport of new ^13^C-labeled soluble carbon compounds to pine tissues. Moreover, mistletoe removal could also lower the sap flow rate of the whole branch (Zweifel et al., 2012), leading to a reduction of carbon assimilates refixation in xylem tissues and further in needles and phloem (Figures 4A–C).

## Conclusion

Our results demonstrate that pine mistletoes are fully carbon-autonomous: they do not provide carbon to the host and are also not supplied with carbon compounds by the host. We also observed that mistletoes are constrained in their photosynthesis by the host when soil water availability is low, most likely due to competition for xylem water—when the competition is released by removing the host needles, the ^13^C assimilation of the mistletoe increases. This result provides physiological evidences that mistletoes do increase the drought stress of their hosts, resulting in an increased mortality risk during severely dry periods previously proposed. We, therefore, conclude that the hemiparasites live on their own in terms of carbon gain which, however, depends on the water provided by the host tree.

## Data Availability Statement

The raw data supporting the conclusions of this article will be made available by the authors, without undue reservation.

## Author Contributions

M-HL, AG, and AR planned the labeling experiment. M-HL and AW designed the treatment and sampling experiment. AW and ML conducted the field work. AW and ZD conducted the experiment work and analyzed the data. AW wrote the manuscript. M-HL, AG, AR, ML, and MS revised the manuscript. All authors contributed to the article and approved the submitted version.

## Conflict of Interest

The authors declare that the research was conducted in the absence of any commercial or financial relationships that could be construed as a potential conflict of interest.

## Publisher’s Note

All claims expressed in this article are solely those of the authors and do not necessarily represent those of their affiliated organizations, or those of the publisher, the editors and the reviewers. Any product that may be evaluated in this article, or claim that may be made by its manufacturer, is not guaranteed or endorsed by the publisher.

## References

[B1] Andersen C. P. Nikolov I. Nikolova P. Matyssek R. Haberle K. H. (2005). Estimating “autotrophic” belowground respiration in spruce and beech forests: decreases following girdling. *Eur. J. For. Res.* 124 155–163. 10.1007/s10342-005-0072-8

[B2] Aschan G. Pfanz H. (2003). Non-foliar photosynthesis–a strategy of additional carbon acquisition. *Flora Morphol. Distrib. Funct. Ecol. Plants* 198 81–97.

[B3] Bickford C. P. Kolb T. E. Geils B. W. (2005). Host physiological condition regulates parasitic plant performance: *Arceuthobium vaginatum* subsp. cryptopodum on *Pinus ponderosa*. *Oecologia* 146 179–189. 10.1007/s00442-005-0215-0 16086165

[B4] Brunner I. Pannatier E. G. Frey B. Rigling A. Landolt W. Zimmermann S. (2009). Morphological and physiological responses of Scots pine fine roots to water supply in a dry climatic region in Switzerland. *Tree Physiol.* 29 541–550. 10.1093/treephys/tpn046 19203972

[B5] De Schepper V. Steppe K. (2013). “Tree girdling: a tool to improve our understanding of coupled sugar and water transport,” in *Proceedings of the II International Symposium on Woody Ornamentals of the Temperate Zone*, Gent, 313–320.

[B6] Dobbertin M. Eilmann B. Bleuler P. Giuggiola A. Pannatier E. G. Landolt W. (2010). Effect of irrigation on needle morphology, shoot and stem growth in a drought-exposed *Pinus sylvestris* forest. *Tree Physiol.* 30 346–360. 10.1093/treephys/tpp123 20067912

[B7] Dobbertin M. Mayer P. Wohlgemuth T. Feldmeyer-Christe E. Graf U. Zimmermann N. (2005). The decline of *Pinus sylvestris* L. forests in the Swiss Rhone valley-a result of drought stress? *Phyton* 45:153.

[B8] Dobbertin M. Rigling A. (2006). Pine mistletoe (*Viscum album* ssp. austriacum) contributes to Scots pine (*Pinus sylvestris*) mortality in the Rhone valley of Switzerland. *For. Pathol.* 36 309–322.

[B9] Durand-Gillmann M. Cailleret M. Boivin T. Nageleisen L.-M. Davi H. (2014). Individual vulnerability factors of Silver fir (*Abies alba* Mill.) to parasitism by two contrasting biotic agents: mistletoe (*Viscum album* ssp. abietis) and bark beetles (Coleoptera: Curculionidae: Scolytinae) during a decline process. *Ann. For. Sci.* 71 659–673.

[B10] Escher P. Eiblmeier M. Hetzger I. Rennenberg H. (2004b). Spatial and seasonal variation in amino compounds in the xylem sap of a mistletoe (*Viscum album*) and its hosts (*Populus* spp. and *Abies alba*). *Tree Physiol.* 24 639–650. 10.1093/treephys/24.6.639 15059764

[B11] Escher P. Eiblmeier M. Hetzger I. Rennenberg H. (2004a). Seasonal and spatial variation of carbohydrates in mistletoes (*Viscum album*) and the xylem sap of its hosts (*Populus euamericana* and *Abies alba*). *Physiol. Plant.* 120 212–219. 10.1111/j.0031-9317.2004.0230.x 15032855

[B12] Galiano L. Martínez-Vilalta J. Lloret F. (2011). Carbon reserves and canopy defoliation determine the recovery of Scots pine 4 yr after a drought episode. *New Phytol.* 190 750–759. 10.1111/j.1469-8137.2010.03628.x 21261625

[B13] Gao D. Joseph J. Werner R. A. Brunner I. Zürcher A. Hug C. (2021). Drought alters the carbon footprint of trees in soils—tracking the spatio-temporal fate of 13C-labelled assimilates in the soil of an old-growth pine forest. *Glob. Change Biol.* 27 2491–2506. 10.1111/gcb.15557 33739617 PMC8251913

[B14] Glatzel G. Geils B. (2009). Mistletoe ecophysiology: host–parasite interactions. *Botany* 87 10–15. 10.1111/plb.12638 28960778

[B15] Joseph J. Gao D. Backes B. Bloch C. Brunner I. Gleixner G. (2020). Rhizosphere activity in an old-growth forest reacts rapidly to changes in soil moisture and shapes whole-tree carbon allocation. *Proc. Natl. Acad. Sci. U.S.A.* 117 24885–24892. 10.1073/pnas.2014084117 32958662 PMC7547207

[B16] Le Q. Tennakoon K. U. Metali F. Lim L. B. Bolin J. F. (2016b). Host specific variation in photosynthesis of an obligate xylem-tapping mistletoe *Dendrophthoe curvata* in a Bornean heath forest. *Nordic J. Bot.* 34 235–243.

[B17] Le Q. Tennakoon K. Metali F. Lim L. Bolin J. (2016a). Ecophysiological responses of mistletoe Dendrophthoe curvata (Loranthaceae) to varying environmental parameters. *J. Trop. For. Sci.* 28 59–67.

[B18] Lüttge U. Haridasan M. Fernandes G. W. de Mattos E. A. Trimborn P. Franco A. C. (1998). Photosynthesis of mistletoes in relation to their hosts at various sites in tropical Brazil. *Trees* 12 167–174.

[B19] Marshall J. D. Dawson T. E. Ehleringer J. R. (1994). Integrated nitrogen, carbon, and water relations of a xylem-tapping mistletoe following nitrogen fertilization of the host. *Oecologia* 100 430–438. 10.1007/BF00317865 28306932

[B20] Marshall J. D. Ehleringer J. R. (1990). Are xylem-tapping mistletoes partially heterotrophic? *Oecologia* 84 244–248. 10.1007/BF00318279 28312760

[B21] R Core Team (2021). *R: A Language and Environment for Statistical Computing. Version 3.5.1.* Vienna: R foundation for statistical computing.

[B22] Reich P. B. Sendall K. M. Stefanski A. Rich R. L. Hobbie S. E. Montgomery R. A. (2018). Effects of climate warming on photosynthesis in boreal tree species depend on soil moisture. *Nature* 562 263–267. 10.1038/s41586-018-0582-4 30283137

[B23] Richter A. Popp M. Mensen R. Stewart G. Willert D. (1995). Heterotrophic carbon gain of the parasitic angiosperm *Tapinanthus oleifolius*. *Funct. Plant Biol.* 22 537–544.

[B24] Rigling A. Bigler C. Eilmann B. Feldmeyer-Christe E. Gimmi U. Ginzler C. (2013). Driving factors of a vegetation shift from scots pine to pubescent oak in dry alpine forests. *Glob. Change Biol.* 19 229–240. 10.1111/gcb.12038 23504734

[B25] Rigling A. Eilmann B. Koechli R. Dobbertin M. (2010). Mistletoe-induced crown degradation in scots pine in a xeric environment. *Tree Physiol.* 30 845–852. 10.1093/treephys/tpq038 20504777

[B26] Sangüesa-Barreda G. Linares J. C. Camarero J. J. (2012). Mistletoe effects on scots pine decline following drought events: insights from within-tree spatial patterns, growth and carbohydrates. *Tree Physiol.* 32 585–598. 10.1093/treephys/tps031 22539634

[B27] Sangüesa-Barreda G. Linares J. C. Camarero J. J. (2013). Drought and mistletoe reduce growth and water-use efficiency of Scots pine. *For. Ecol. Manag.* 296 64–73.

[B28] Scalon M. Haridasan M. Franco A. (2013). A comparative study of aluminium and nutrient concentrations in mistletoes on aluminium-accumulating and non-accumulating hosts. *Plant Biol.* 15 851–857. 10.1111/j.1438-8677.2012.00713.x 23452024

[B29] Scalon M. C. Wright I. J. (2015). A global analysis of water and nitrogen relationships between mistletoes and their hosts: broad-scale tests of old and enduring hypotheses. *Funct. Ecol.* 29 1114–1124.

[B30] Scalon M. C. Wright I. J. (2017). Leaf trait adaptations of xylem-tapping mistletoes and their hosts in sites of contrasting aridity. *Plant Soil* 415 117–130.

[B31] Schaub M. Haeni M. Hug C. Gessler A. Rigling A. (2016). *Tree Measurements 2002-2016 From the Long-Term Irrigation Experiment Pfynwald.* Valais. Swiss Federal Research Institute WSL SwissForestLab.

[B32] Schönbeck L. Gessler A. Hoch G. McDowell N. G. Rigling A. Schaub M. (2018). Homeostatic levels of nonstructural carbohydrates after 13 yr of drought and irrigation in *Pinus sylvestris*. *New Phytol.* 219 1314–1324. 10.1111/nph.15224 29770969

[B33] Schonbeck L. Grossiord C. Gessler A. Gisler J. Meusburger K. D’Odorico P. (2021). Photosynthetic acclimation and sensitivity to short-and long-term environmental changes. *bioRxiv* [Preprint] 10.1101/2021.01.04.425174 35134157

[B34] Schulze E. D. Turner N. Glatzel G. (1984). Carbon, water and nutrient relations of two mistletoes and their hosts: a hypothesis. *Plant Cell Environ.* 7 293–299.

[B35] Simkin A. J. Faralli M. Ramamoorthy S. Lawson T. (2020). Photosynthesis in non-foliar tissues: implications for yield. *Plant J.* 101 1001–1015. 10.1111/tpj.14633 31802560 PMC7064926

[B36] Smith P. L. Gledhill D. (1983). Anatomy of the endophyte of *Viscum album* L.(Loranthaceae). *Bot. J. Linn. Soc.* 87 29–53.

[B37] Solomon N. U. James I. M. Alphonsus N. O.-O. Nkiruka R. U. (2015). A review of host-parasite relationships. *Annu. Res. Rev. Biol.* 5 372–384.

[B38] Streicker D. G. Fenton A. Pedersen A. B. (2013). Differential sources of host species heterogeneity influence the transmission and control of multihost parasites. *Ecol. Lett.* 16 975–984. 23714379 10.1111/ele.12122 PMC4282463

[B39] Těšitel J. Plavcová L. Cameron D. D. (2010). Interactions between hemiparasitic plants and their hosts: the importance of organic carbon transfer. *Plant Signal. Behav.* 5 1072–1076. 10.4161/psb.5.9.12563 20729638 PMC3115071

[B40] Vacchiano G. Garbarino M. Mondino E. B. Motta R. (2012). Evidences of drought stress as a predisposing factor to scots pine decline in Valle d’Aosta (Italy). *Eur. J. For. Res.* 131 989–1000.

[B41] Wang J. R. Hawkins C. Letchford T. (1998). Photosynthesis, water and nitrogen use efficiencies of four paper birch (*Betula papyrifera*) populations grown under different soil moisture and nutrient regimes. *For. Ecol. Manag.* 112 233–244.

[B42] Yan C. F. Gessler A. Rigling A. Dobbertin M. Han X. G. Li M. H. (2016). Effects of mistletoe removal on growth, N and C reserves, and carbon and oxygen isotope composition in scots pine hosts. *Tree Physiol.* 36 562–575. 10.1093/treephys/tpw024 27083524 PMC4886294

[B43] Zuber D. (2004). Biological flora of central Europe: *Viscum album* L. *Flora Morphol. Distrib. Funct. Ecol. Plants* 199 181–203.

[B44] Zweifel R. Bangerter S. Rigling A. Sterck F. J. (2012). Pine and mistletoes: how to live with a leak in the water flow and storage system? *J. Exp. Bot.* 63 2565–2578. 10.1093/jxb/err432 22268160

