# Supplementary figures and images for: There Is No Carbon Transfer Between Scots Pine and Pine Mistletoe but the Assimilation Capacity of the Hemiparasite Is Constrained by Host Water Use Under Dry Conditions
Source: Front Plant Sci. 2022 May 26;13:902705. doi: 10.3389/fpls.2022.902705 (PMC9201984; doi:10.3389/fpls.2022.902705)

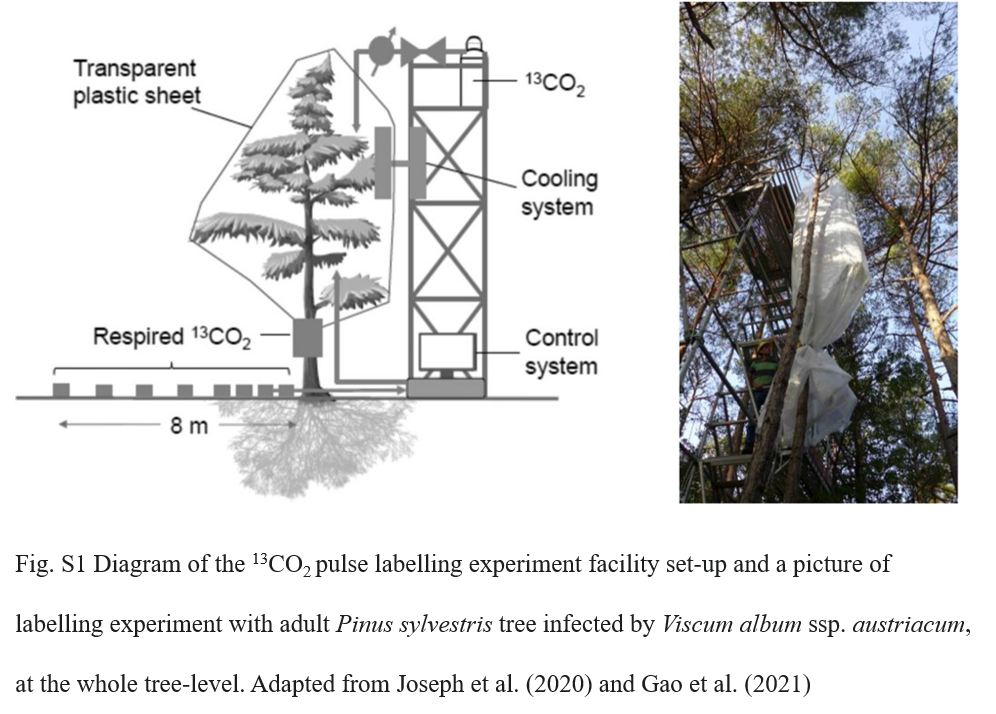

Supplement: Supplementary file 1 [file Image_1.PNG]

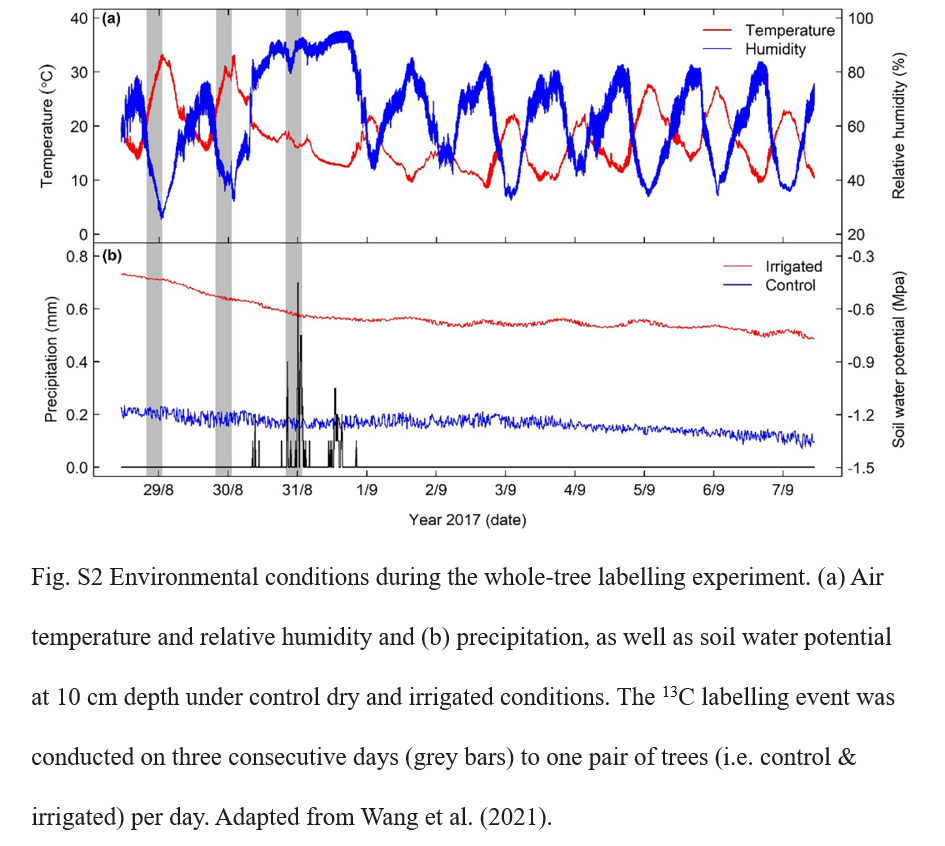

Supplement: Supplementary file 2 [file Image_2.PNG]
